# Analysis and novel methods for capture of normative eye-tracking data in 2.5-month old infants

**DOI:** 10.1371/journal.pone.0278423

**Published:** 2022-12-09

**Authors:** Alma Gharib, Barbara L. Thompson

**Affiliations:** 1 Department of Computer Science, University of Southern California, Los Angeles, California, United States of America; 2 Program in Developmental Neuroscience and Neurogenetics, The Saban Research Institute and Department of Pediatrics at Children’s Hospital of Los Angeles, Keck School of Medicine, University of Southern California, Los Angeles, California, United States of America; 3 Department of Pediatrics and Human Development, College of Human Medicine, Michigan State University, Grand Rapids, Michigan, United States of America; The Ohio State University, UNITED STATES

## Abstract

Development of attention systems is essential for both cognitive and social behavior maturation. Visual behavior has been used to assess development of these attention systems. Yet, given its importance, there is a notable lack of literature detailing successful methods and procedures for using eye-tracking in early infancy to assess oculomotor and attention dynamics. Here we show that eye-tracking technology can be used to automatically record and assess visual behavior in infants as young as 2.5 months, and present normative data describing fixation and saccade behavior at this age. Features of oculomotor dynamics were analyzed from 2.5-month old infants who viewed videos depicting live action, cartoons, geometric shapes, social and non-social scenes. Of the 54 infants enrolled, 50 infants successfully completed the eye-tracking task and high-quality data was collected for 32 of those infants. We demonstrate that modifications specifically tailored for the infant population allowed for consistent tracking of pupil and corneal reflection and minimal data loss. Additionally, we found consistent fixation and saccade behaviors across the entire six-minute duration of the videos, indicating that this is a feasible task for 2.5-month old infants. Moreover, normative oculomotor metrics for a free-viewing task in 2.5-month old infants are documented for the first time as a result of this high-quality data collection.

## Introduction

Eye movements subserve visual function and cognition, and gaze behaviors have been called a “window into the mind” of an observer [1]. Early in development when infants cannot be instructed to complete a task, or to speak or respond verbally, passive measurement of gaze behavior has been demonstrated to be a robust and reliable method for assessing both typical and atypical brain and cognitive function [2–4]. However, in order to use visual behavior as a tool to assess cognitive function, it is first necessary to characterize normative oculomotor behavior in early infancy.

Precise measurement of eye movements such as fixations and saccades provides valuable information regarding how visual attention is deployed. Fixations are periods when the eye remains relatively still and is receiving visual input [5, 6]. This metric has been used to gain insight into how humans engage with stimuli and process visual information. Saccades are rapid eye movements that shift the point of fixation. During saccadic movements, most forms of visual input are suppressed [7–9]. Saccades provide insight into the ability to shift attention, and the capacity to search for and integrate visual information. Comparison of normative and aberrant fixation and saccade data has been used to detect and characterize attentional and developmental disorders such as autism spectrum disorder (ASD) [10, 11], attentional deficit hyperactivity disorder (ADHD) [12, 13], fetal alcohol spectrum disorder (FASD) [14], and schizophrenia [12, 15].

**Fig 1:**
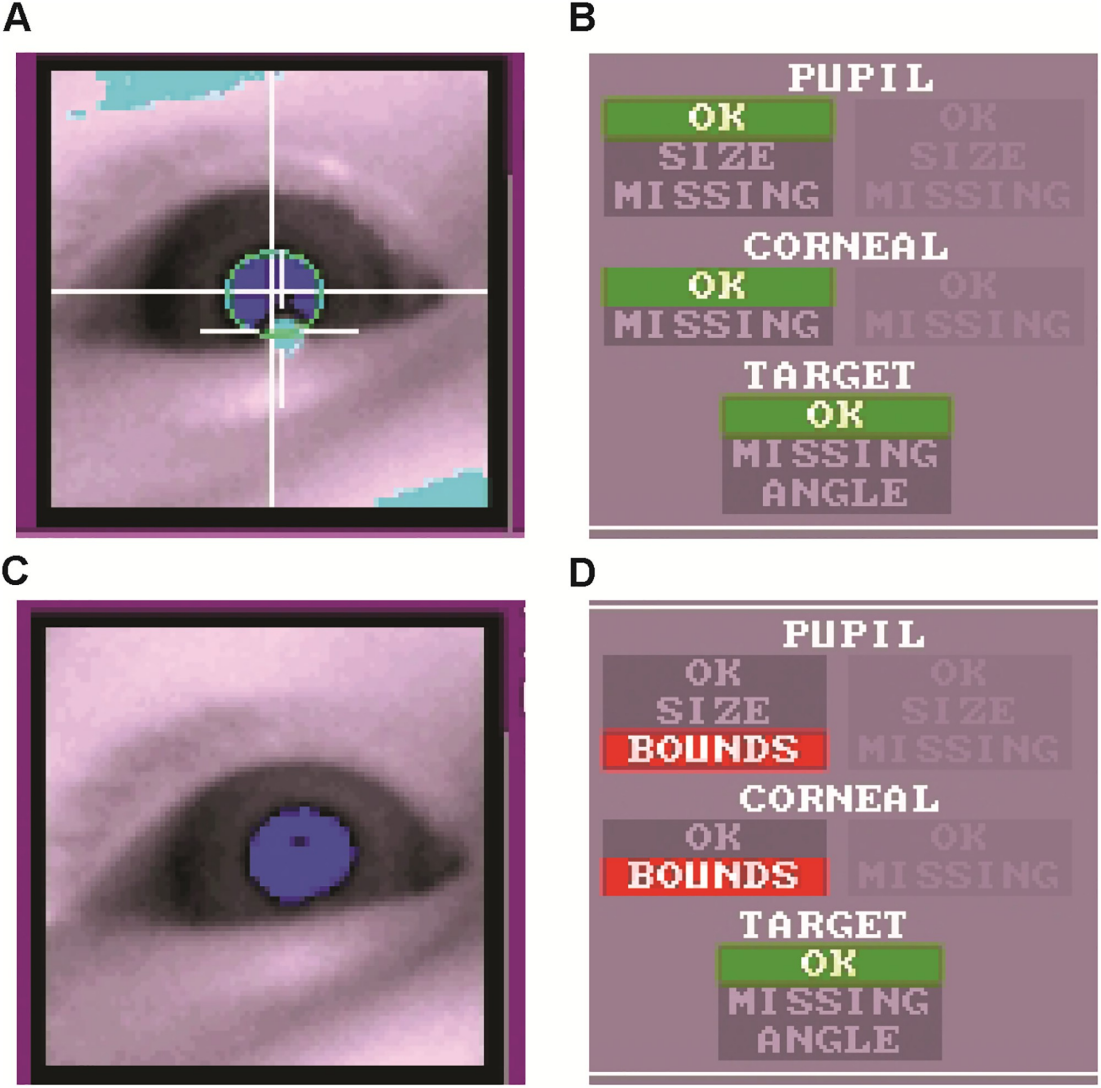
Example of loss of pupil and corneal reflections. A participant whose corneal and pupil reflection were initially successfully tracked (A,B), then subsequently obstructed by the lower eyelid (C,D). The Eyelink system tracks head distance relative to the camera using a small target sticker placed on the infant’s forehead. If the infant shifts position or turns away from the camera, tracking of the target sticker can be lost. The eye-tracker view of the participant’s eye image (A) demonstrates that pupil reflection (dark blue) and corneal reflection (cyan) were initially tracked by the camera. Status indicators (B) confirm that the camera was tracking the target sticker in addition to the pupil and corneal reflections. Relatively small head movements resulted in loss of pupil and corneal reflection due to obstruction by the lower eyelid (C). Status indicators (D) confirmed loss of pupil and corneal reflection, but no obstruction of the target sticker, verifying that loss of corneal reflection is not due to significant head movement or the infant looking away from the camera.

While a large body of existing literature outlines methodologies for collecting and examining visual behavior in adults and older children, collecting eye-tracking data in infants presents a unique set of additional technical challenges. Current methodological impediments include physical limitations inherent to infant development that affect data collection and quality. First, there is a lack of muscular control of the neck in children under 6 months of age. Infants below this age cannot consistently sit upright unassisted, nor maintain control over their head position. This results in a high degree of head movement, not to mention discomfort for the infant if seated upright, that disrupts eye tracking data collection. Second, infants’ facial and eye structure can pose challenges for establishing a clear line of sight for the camera and light source which are both positioned below the participant’s face. While chubby infant cheeks are known to create a cuteness perception in adults, thereby enhancing the survival of the infant [16], they can also interfere with infrared eye-tracking techniques by obstructing the infrared light source. Similarly, the physical structure of an infant’s lower eyelid also can interfere with tracking of the corneal reflection due to the greater volume of periorbital fat in early infancy. At younger ages, the anterior surround of the eye socket is mainly filled with fat which can occlude the corneal reflection, impeding its detection. As the infant continues to develop, the fat migrates posteriorly while the eye is pushed forward [17]. Third, the physical size of an infant’s eyeball is smaller than an adult’s [17], and there is a smaller distance between the top and lower eyelids [18]. Thus, any downward movement of the eye when the infant gazes down, or as occurs when the infant is sleepy, will increase signal loss more so than in adults (Fig 1). Fourth, the ability of the infrared light source to illuminate the pupil and cornea can be impeded by both transient and stable factors related to the appearance of the eye, such as watery eyes immediately following crying or from a cold, or light pigmentation of the iris as is common in newborns [19]. Finally, the majority of a newborn’s time is spent in sleep states, with as little as 20% in alert states [20]. Therefore, a task with a quick initial setup and minimal demands on the infant is necessary for successful data collection in early infancy.

There are numerous studies investigating visual behavior in older infants under the age of 2 years (see [21] for a review). For example, researchers have probed visual attention in infancy using static stimuli [22, 23], perception/discrimination of specific stimulus properties [23–28], fixation behavior using structured tasks [25, 27, 28], and investigated broad, global measures of looking preferences or gaze time [29–32]. Similar to the present study, researchers have also investigated infant visual behavior during free-viewing using dynamic stimuli [10, 33, 34]. Findings from these studies have advanced our understanding of critical dimensions of infant attention and looking preferences. However, there remains a gap in quantifying the basic oculomotor metrics in young infants during a free-viewing task.

Given the difficulties with eye-tracking in very young infants, it comes as no surprise that there are few studies using infants at 6 months of age or below [10, 35, 36]. Some studies that have collected data at younger than 6 months have relied on highly-specialized eye-tracker and participant configurations that allow the eye-tracker and stimulus screen to be suspended in air above the child while the child reclines in a commercial car seat or baby seat [10, 26]. This adaptation in a laboratory setting ameliorates the physical challenges noted above, but limits the portability of the system. Other studies in young infants have skipped calibration or validation procedures entirely making it difficult to assess accuracy of the data collected [24]. The current methodological investigation was designed to adapt routine eye-tracking techniques commonly used in older children and adults for capturing high quality eye-tracking data in very young infants, to determine data quality thresholds, and to precisely characterize oculomotor fixation and saccade behavior in 2.5-month old infants. Methodological success would address current knowledge gaps of oculomotor functioning in very young infants, as well as create opportunities for applying a scalable protocol to acquire high quality data in young infants.

## Methods

### Participants

Fifty-seven mother-infant dyads were enrolled in a larger longitudinal study investigating resiliency to early adversity when infants were approximately two and a half months old. Eligible participants were recruited from the AltaMed Health Services pediatric clinic located at Children’s Hospital Los Angeles. Women who had recently given birth and were receiving early postnatal services were presented with informational flyers about the study. If mothers indicated interest, they were provided with further information about the research study. Participants were excluded if the child (i) was born prematurely (<36 gestational weeks), (ii) had low birth weight (< 2500 grams), (iii) had identified genetic, metabolic, syndromic or progressive neurological disorders (including epilepsy, Down Syndrome, Rett Syndrome, Tuberous Sclerosis, Neurofibromatosis, Fragile X Syndrome), (iv) had congenital malformations, surgical interventions, (v) had severe sensory or motor impairments (deafness, blindness) or, (vi) if mothers experienced maternal preeclampsia, or perinatal birth complications (e.g. NICU stay beyond 2 days). Exclusion criteria were confirmed through pre-enrollment screening, though 3 infants were enrolled due to inaccurate pre-enrollment screening. These participants were excluded from analysis, bringing the adjusted number of enrolled participants to 54 mother-infant dyads. This study was conducted in accordance with the ethical standards of the American Psychological Association. Ethical approval for the study was obtained from Children’s Hospital Los Angeles, CHLA-15-00267, Resiliency to Toxic Stress. Informed written consent in the mothers preferred language (English or Spanish) was obtained from the mother for all infant-mother dyads who participated in the study.

### Equipment

Visual stimuli were presented on a 24-inch computer monitor with a native resolution of 1920 x 1200 pixels and refresh rate of 60 Hz. Eye-movements were recorded via a non-invasive and contactless infrared Eyelink 1000 (SR Research, Osgoode, ON, Canada) eye-tracking system in remote mode. The monitor was mounted on a flexible arm, with the eye-tracking camera and infrared illuminator unit affixed on a bracket extending 11cm from the bottom edge of the monitor. For infant data collection, the standard 890nm infrared illuminator was replaced with a longer-wavelength 940nm illuminator and 16mm lens (SR Research). Corneal and pupil reflection were recorded at a sampling rate of 500 Hz (with a spatial resolution less than 0.5°). Stimuli were presented at a resolution of 1280 x 720 pixels, at a rate of 30Hz. Visual scenes subtended approximately a 32° (horizontal) by 18° (vertical) visual angle at a distance of 60cm.

### Stimuli

**Fig 2:**
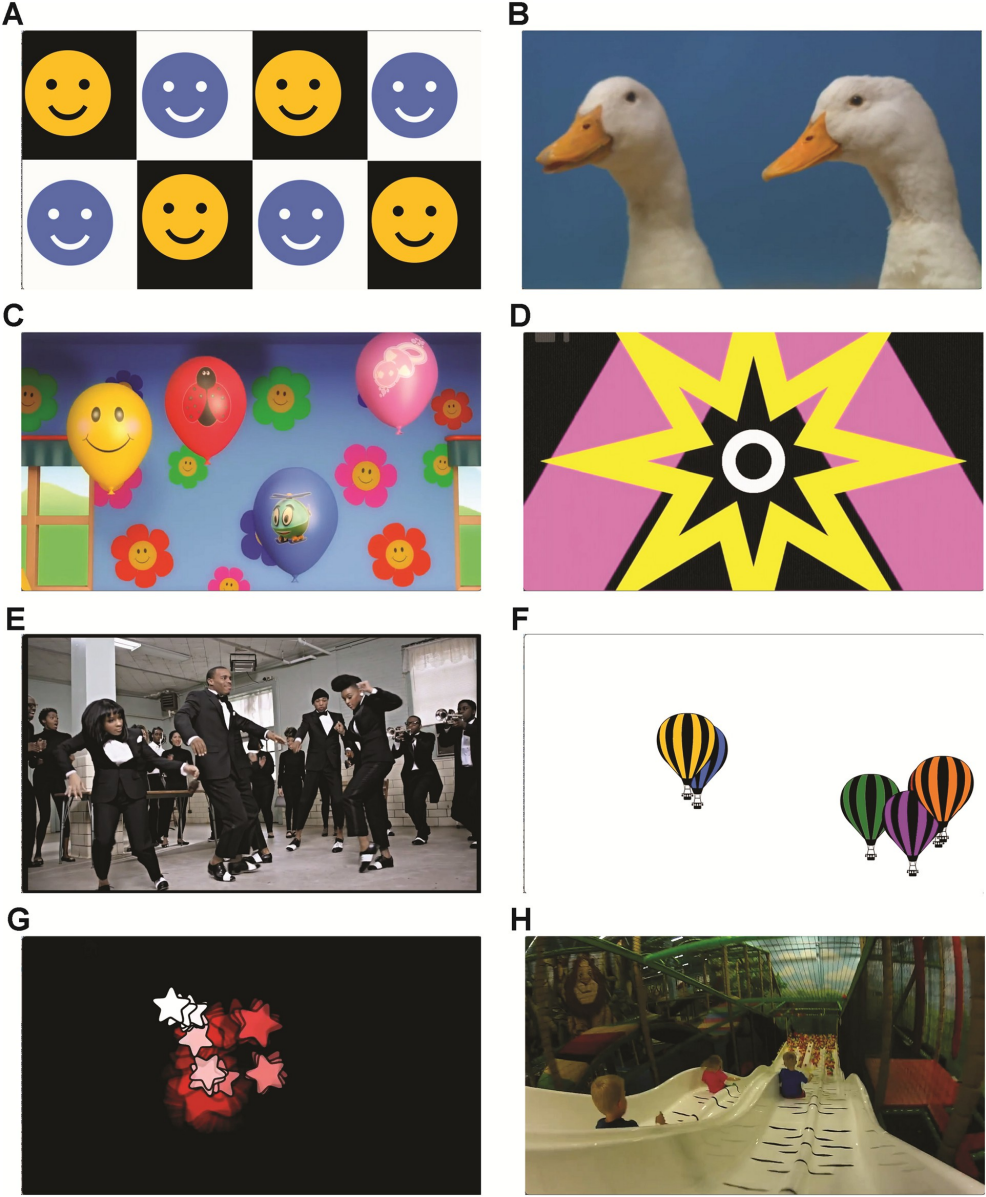
Static images of video stimuli.

Calibration was performed using a 3-point triangular calibration procedure at the beginning of each recording session. A custom-animated precessing dog with accompanying barking sound set on a solid mid-gray (180R x 180G x 180B) background was used as the calibration target. For the data collection stimuli, an assortment of child-friendly videos were selected from YouTube. Videos consisted of live action scenes, animation, animals, moving shapes and graphics, and social and non-social scenes. Static images of select video scenes are shown in Fig 2. Scenes that were 2–4 seconds in length were extracted and edited together to create blocks that were 50–55 seconds long, containing 15 clips per block. No scenes were repeated in the study. Trial order was pseudo-randomized such that the first 3 trials always consisted of blocks A, B, and C, in randomized order and the second 3 trials always consisted of blocks D, E, and F, also in randomized order for a total of 6 blocks presented across approximately 6 minutes. Sound from the original videos was included, but none of the clips contained discernible language. Each trial began with a precessing annulus accompanied by audio of a quacking duck to attract and maintain the infant’s attention towards the screen before the video stimuli began playing. Experiment Builder software (SR Research) was used for stimulus presentation and data collection.

Video clips contained animals, moving shapes and graphics, and live action scenes. Video scenes were varied in the amount of movement, the directionality of movement, and the speed of stimulus movement.

### Procedure

**Fig 3:**
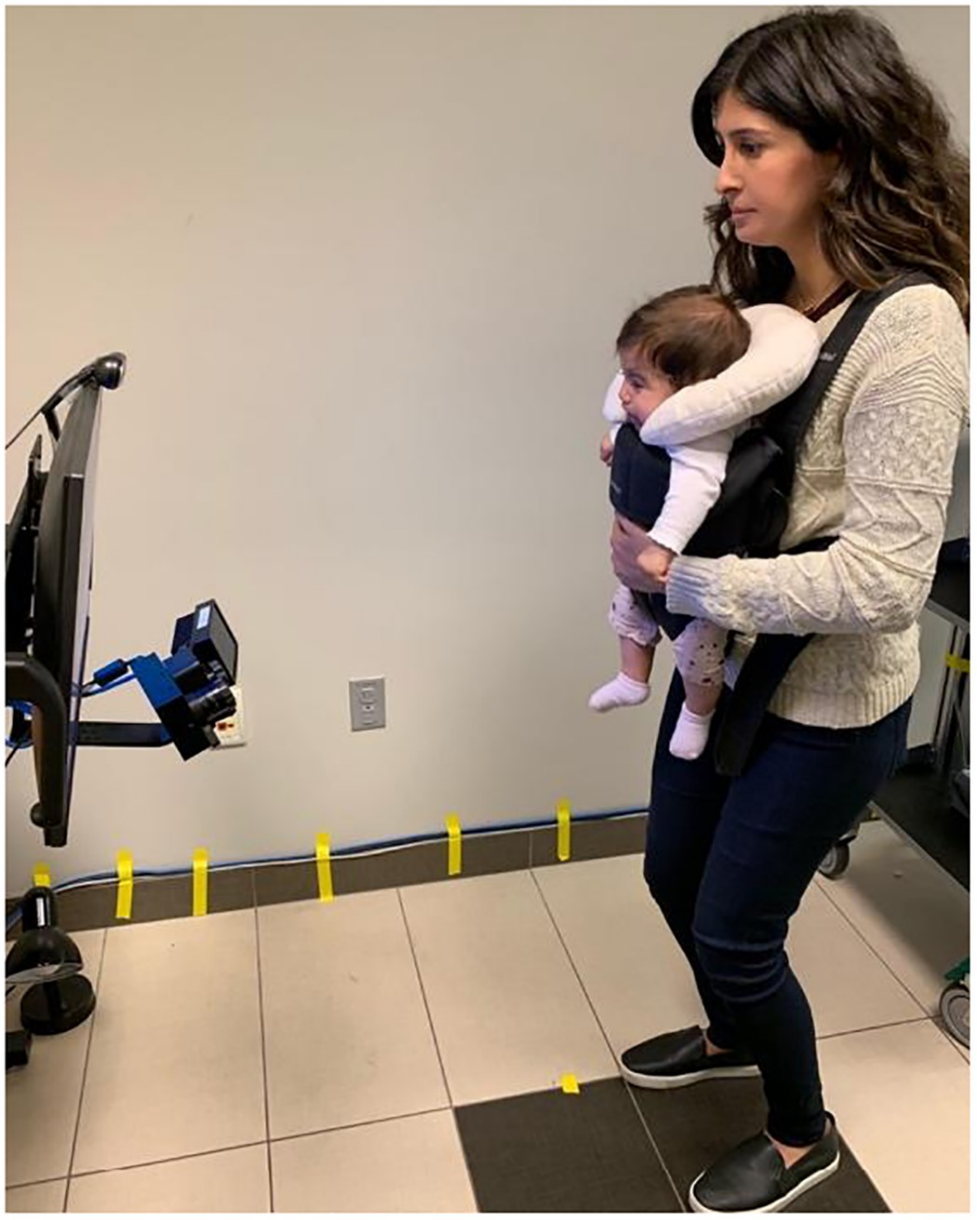
Modifications to standard eye-tracking setup.

The research visit took place in a child-friendly testing room with ample seating for families. A member of the research team explained the study, asked the parents if they had questions, and then consented the mother-infant dyad for participation in the study. Caregivers were instructed they could stop, take a break, or discontinue the study at any point. Mothers then placed their infant on the changing table so the researcher could interact with and become familiar with the infant. The mother was invited to remain in the testing space for the duration of the experiment and was asked to remain quiet and out of the infants view so as to not distract the infant’s attention away from the visual stimuli. Once the infant was familiarized with the researcher, the infant was placed into a commercial infant carrier (BabyBjorn carrier original, 2010) worn by the researcher, and a commercial infant pillow (Baby Moon Pod) was placed between the researcher’s chest and the back of the infant’s head (Fig 3). Extraneous auditory and visual stimuli were minimized during the duration of data collection.

The infant was secured in a commercial baby carrier held by a member of the research team, facing outward with an infant neck pillow placed behind the head for stabilization of the head. The stimulus monitor and eye-tracking camera were affixed to a moveable arm mount, with the infant positioned at a distance of approximately 60cm from the camera. These adjustments were necessary to minimize loss of corneal reflection and movements due to neck instability.

A black and white target sticker was placed in the middle of the infant’s forehead. This sticker was used to measure and record infant head distance to the camera in real-time by the eye-tracking software. The experimenter held the infant facing forward towards the stimulus monitor, with the infant’s forehead at a distance of 570–600 mm away from the eye-tracking camera. A second researcher was also present during the eye-tracking to monitor visual gaze as it was recorded by the eye-tracker and to perform troubleshooting and redirection of the infant’s attention if necessary. If during the calibration routine or stimulus presentation the infant’s attention wandered away from the stimulus monitor, the second researcher used a squeaky toy to re-direct the infant’s attention to the visual stimulus. Audio feedback from the eye-tracker (i.e. an audible “lost track” beeping noise) indicated a loss of signal tracking, and provided the experimenter with knowledge on whether the infant needed repositioning or redirecting of visual gaze. After calibration was deemed successful, or when the infant started losing attention, the experiment was initiated, resulting in a few participants skipping calibration. Eye-tracking always included attempts to secure binocular data, though data from only one eye was used for analyses. The entire task lasted approximately 7 minutes (1 minute for calibration and 6 minutes for stimuli presentation).

### Data processing and analysis

**Fig 4:**
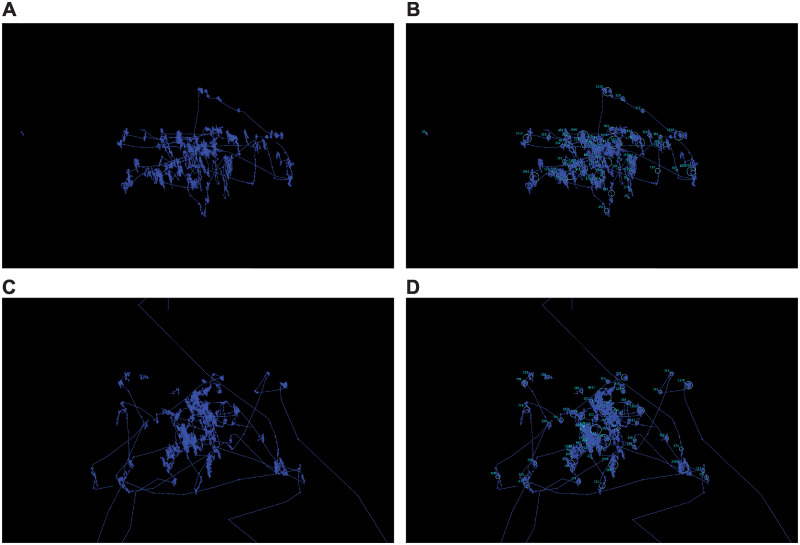
Sample level gaze data. Participant gaze data from two different trials (A,B and C,D) are shown. Gaze positions in screen coordinates are shown in A and C. Fixation durations are indicated numerically next to Eyelink identified parsed fixations which are shown as light blue circles in B and D.

EyeLink 1000 EDF data files were converted using the Edf2Mat Matlab Toolbox [37]. The Edf2Mat Matlab Toolbox was modified in-house and used for the first iteration of processing to extract fixation and saccade events directly from EDF files (Fig 4). Further processing to trim and clean data, including thresholding and low-pass filtering as described in further detail below, was performed using a pipeline of custom Matlab scripts.

A number of parameters were imposed for data analysis. First, since trials were not equal in duration (ranging from 52 to 55 seconds), only the last 50 seconds of data in each trial were used for data analyses. Second, fixation and saccade events were retained only when the start and end times for the event were both contained within the 50 second window. Third, data points in the time series were defined as ‘valid’ when an x,y gaze position coordinate was recorded by the tracker at a given time point. Individual trials were examined and identified as having valid data using a threshold of 70%. Trials were excluded if less than 70% of the duration of the trial contained ‘valid’ data. Data from participants that did not have 6 trials meeting or surpassing the 70% threshold were excluded from analysis of oculomotor dynamics. Fourth, low-pass filters were implemented to remove physiologically-implausible data and to ensure data fidelity. Individual fixation and saccade events in a trial were excluded if they exceeded the following cutoffs: Fixation duration > 1500 milliseconds, saccade duration > 300 milliseconds, saccade mean velocity > 1000 degrees/second, saccade amplitude > 25 degrees. These cutoffs are thresholds that are outside the ranges of what are considered plausible values for fixation [38–41] and saccade parameters [40, 42–45] and were used to reduce noise from the sample.

If binocular data was collected, data from the eye that had higher calibration quality classification as reported by the built-in calibration and validation routine in Experiment Builder was used for analysis. The general average error ranges in degrees used to classify calibration quality by the software were: Good (< 1 degree error), Fair (1–1.5 degrees error), Poor (> 1.5 degrees error). In the case of binocular data collection where calibration classification was equal for both eyes, the left eye was selected for analysis. As a result of the eye selection algorithm, all data reported in the results are from the left eye.

Fixation metrics that were analyzed included fixation duration and count. Saccade metrics that were analyzed included saccade duration, count, average velocity and amplitude. Percentage of valid data and individual oculomotor dynamics across trials were analyzed using a repeated-measures ANOVA, with a within-subject factor of trial number. Statistical analyses were performed using SPSS Statistics for Mac, Version 25 (IBM Corp, Armonk, NY). Degrees of freedom were Greenhouse-Geisser corrected where violations of sphericity occurred.

## Results

### Participants

**Fig 5:**
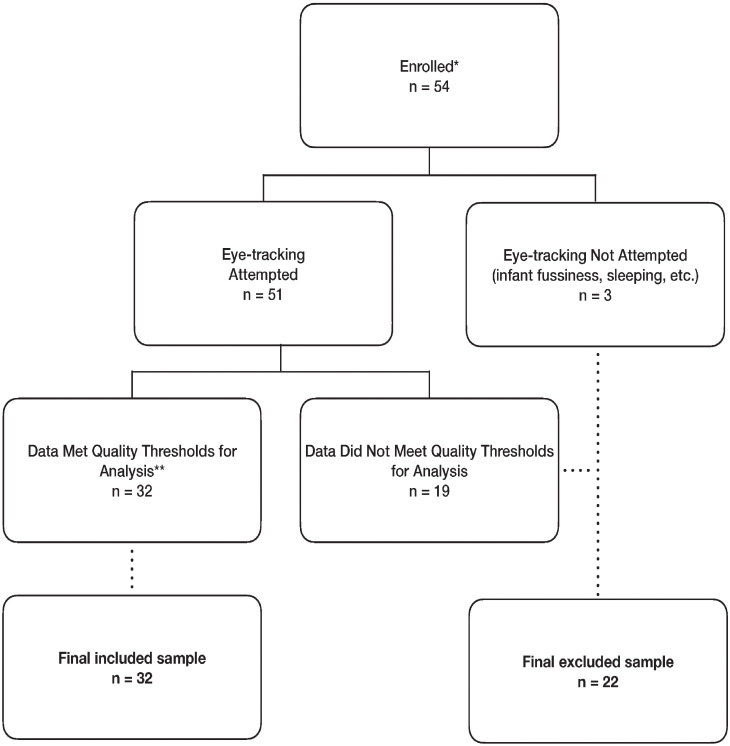
Participant enrollment and data analysis flow chart. A total of 54 mother-infant dyads were enrolled. *An additional 3 dyads were excluded from analysis due to inaccurate pre-enrollment screening. Eye-tracking was not attempted for 3 participants due to infant temperament and difficulty establishing a reliable corneal reflection. For the 51 participants for whom eye-tracking was attempted, data quality metrics are reported. Data from 32 of the 51 participants met or surpassed the 70% threshold for valid data across all 6 trials, and are reported in the analysis of oculomotor dynamics. ** For 2 of the 32 participants whose data met quality thresholds, their data was excluded from analysis of saccade amplitude and saccade velocity due to an error in screen resolution affecting those specific calculations, but was retained for all other analyses of saccade and fixation metrics.

A total of 54 infant participants (n = 23 females) were enrolled with a mean (*M*) age of 2.49 months with standard deviation (*SD*) of 0.36 months. Of the 54 enrolled participants, eye-tracking was not attempted on 3 infants due to the infant being asleep (n = 1), or difficulty establishing a reliable and stable corneal reflection (n = 2). Eye-tracking data was collected from a total of 51 infants, with all but 1 completing the task (infant became fussy mid-task so the task was discontinued). Of the 50 infants who completed the eye-tracking task, 32 participants met or surpassed the 70% valid data threshold for all 6 trials and were used for analyses of oculomotor dynamics. The ages of the 32 infant participants (n = 12 females) meeting 70% threshold were *M* = 2.6 months, *SD* = 0.39 (Table 1). Lastly, for 2 of the 32 participants whose data met quality thresholds, their data was excluded from analysis of saccade amplitude and saccade velocity due to a programming error in screen resolution during data collection that was detected and resolved for subsequent participants (Fig 5).

**Table 1:** Demographic information for included and excluded participants.

	Included (n = 32)	Excluded (n = 22)	p
Child Age (months)	2.6	2.3	.007
Maternal Age (years)	29.6	28.5	> .05
Sex	12 F; 20 M	11 F; 11 M	> .05
Birthweight (grams)	3426	3307	> .05

### Data quality and choice of threshold

#### Calibration quality and binocular/monocular recording rates

**Fig 6:**
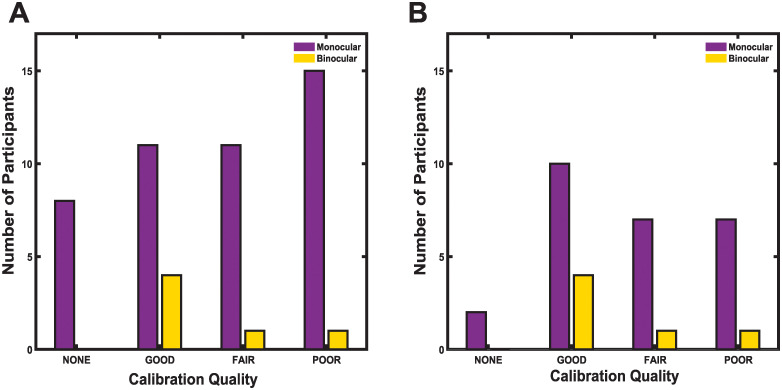
Validation quality and monocular and binocular success rates. Data is shown for participants for whom (A) eye-tracking was attempted (n = 51) and (B) whose data met quality thresholds for analysis of oculomotor metrics (n = 32). Although monocular data was used for analyses, binocular recording was attempted for all participants to maximize the probability of collecting high quality data from at least one eye. If calibration was repeated, only the final attempt is reported here. Infants for whom calibration was aborted or skipped after multiple unsuccessful attempts are represented by the “None” category.

**Fig 7:**
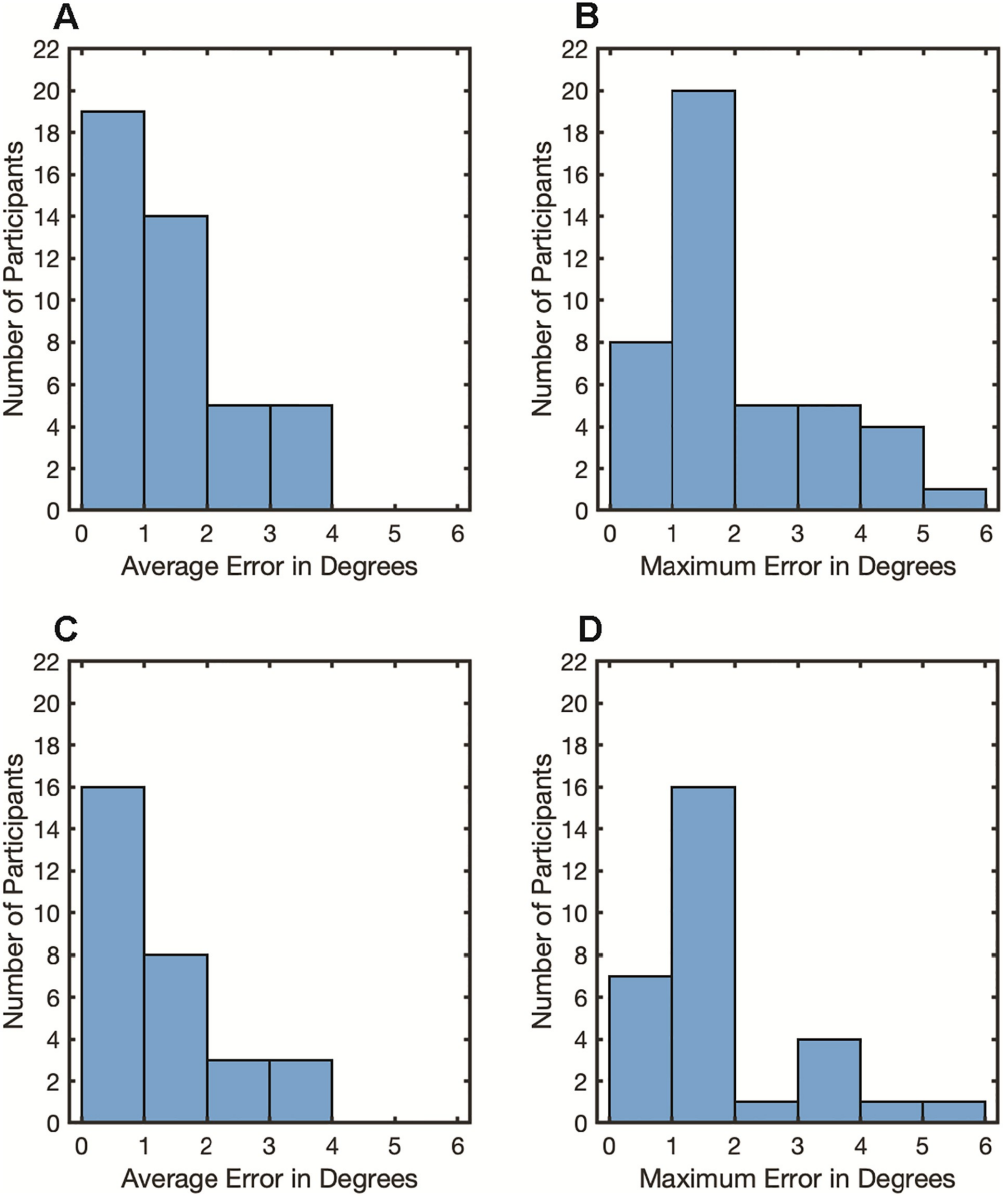
Histograms of calibration and validation error. Average error and maximum error during the calibration and validation procedure for the participants for whom eye-tracking was attempted and calibration was obtained (n = 51) (A, B) and participants whose data met data quality thresholds for subsequent analysis of oculomotor metrics (n = 32) (C, D). Error values indicate the amount of offset between the center of the calibration graphic and the reported x,y coordinates of the gaze sample. If monocular data was collected, values are reported for the recorded eye. If binocular data was collected, values are reported for the eye with higher calibration quality.

Although binocular recording was attempted in all participants (n = 51), binocular data was successfully collected in only 6 participants. For those 6 participants, validation quality was good for 4, fair for 1, and poor for 1. The remaining 45 participants’ data were collected using the monocular setting. Of the 45 monocular recordings, 11 met criteria for good quality, 11 met criteria for fair quality, 15 had poor quality and 8 had calibration routines that were either skipped or aborted after multiple unsuccessful attempts (Fig 6). For the 51 participants for whom eye-tracking was attempted, 27 participants (> 50%) demonstrated an average error that was less than 1.5 degrees (Fig 7; Table 2). While binocular data was collected when possible, data reported were collected from the left eye (refer to Methods for details of eye selection).

**Table 2:** Qualitative output results from calibration and validation procedures.

Calibration Quality	Average Error Range (degrees)	# of Participants
Unsuccessful	-	8
Poor	> 1.5	16
Fair	1–1.5	12
Good	0–1	15

#### Data gaps and selection of a data quality threshold

**Fig 8:**
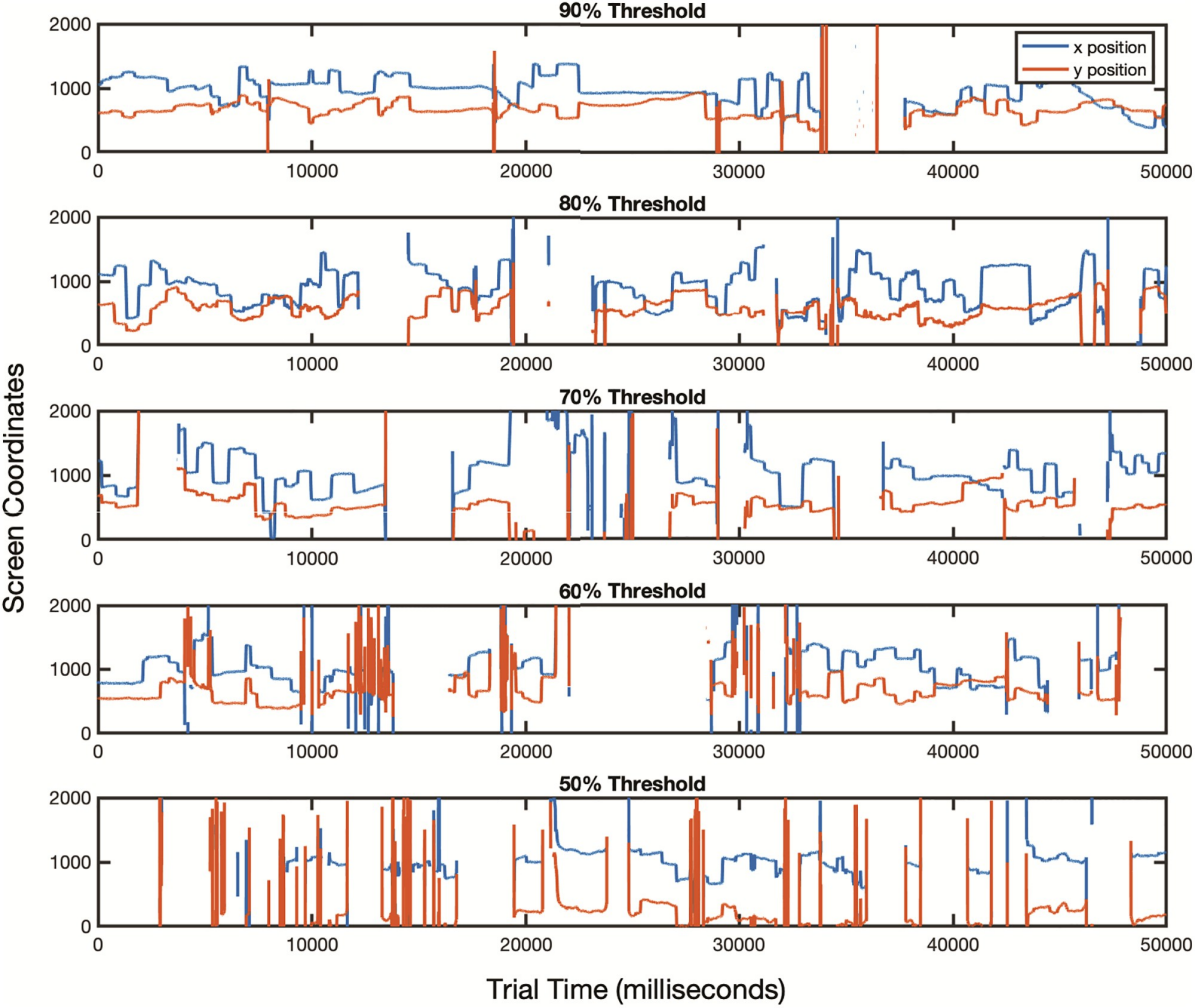
Sample trials of raw data. Raw data are shown from different participants for whom decreasing levels (from top to bottom) of valid data were collected. X and Y eye positions are shown in blue and orange, respectively. Horizontal plateaus are periods where x and y coordinates of the eye remain relatively steady, indicating fixations. Vertical lines are periods where the eye position is changing rapidly, indicating saccades. Gaps in the traces represent periods of data loss. As the threshold for valid data decreases, there are more frequent gaps in the data and increased noise in the x,y traces.

**Fig 9:**
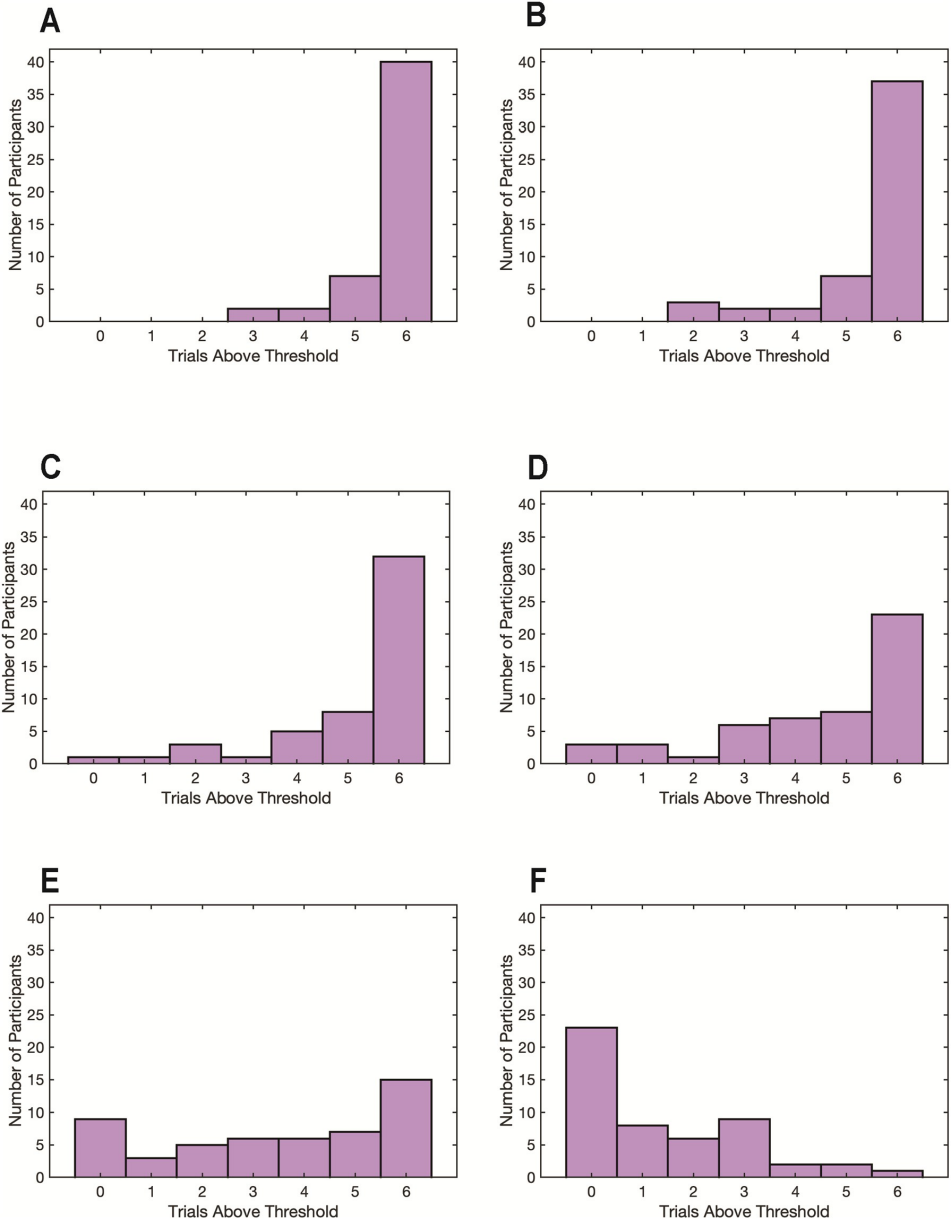
Histograms of different valid data thresholds. The number of participants and number of trials that met or exceeded cutoffs for valid data at 50 (A), 60 (B), 70 (C), 80 (D), 90 (E) and 99 (F) percent thresholds are shown.

For determining data quality, we calculated the number of time points in each trial for which an XY coordinate was collected divided by the number of time points in the trial. This percentage was referred to as the data quality metric which we used to implement a threshold. To determine an appropriate threshold level in the current study of young infants as there is no universally agreed-upon threshold, we examined the impact of 5 different threshold levels of missing data and their effect on outcome variables by conducting a visual inspection of a random sampling of trials (Fig 8). With a high threshold (90% valid, 10% missing), longer fixations and fewer saccades are observed, consistent with extant literature [46, 47]. As the threshold value decreases, fixations become shorter and the number of saccades increase due to noise artifacts interrupting the data stream or gaps due to missing gaze coordinates in the data stream. In the present study and analyses, we selected a threshold of 70% to maximize the number of participants for whom we could compare data across all 6 trials, allowing us to retain approximately 60% (32 of 54 participants) for further analysis. For comparison, if an 80% threshold were implemented, as is often used in adult eye-tracking studies, it would yield retention of 23 of 54 participants’ data (Fig 9). From examination of the data, the selected threshold of 70% provided a reasonable cutoff, with little excess signal noise or data loss in the x,y traces. In addition, most of the horizontal plateaus indicating fixations were preserved (Fig 8).

#### Percentage of valid data

**Fig 10:**
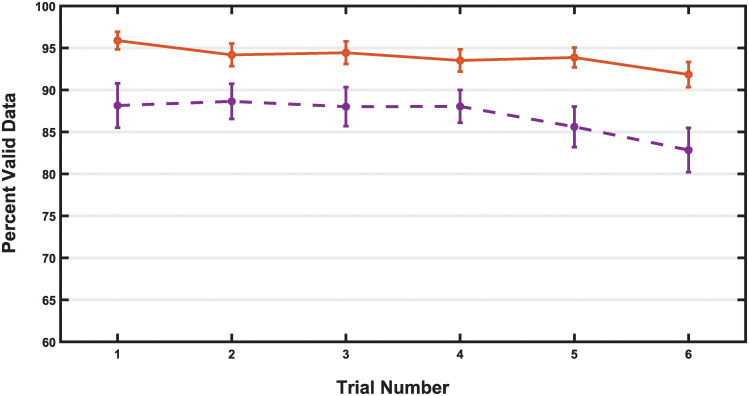
Percent of valid data in thresholded and unthresholded trials. The percentage of valid data across thresholded (orange) and unthresholded (purple) trials is shown. The unthresholded trials include data from all 50 participants who completed the eye-tracking task. Thresholded trials include the 32 participants whose data met or exceed 70% valid data across all 6 trials. In this population of young infants, there was no statistically significant loss of valid data across time in either the thresholded or unthresholded trials. Data presented as mean +/- 1 standard error of the mean (SEM).

To investigate the effect of applying a threshold on percentage of valid data over time, we also analyzed the percentage of valid data for all participants who completed the eye-tracking task (n = 50) regardless of data quality (Fig 10) across trials. We found there was no significant loss of data across time (i.e. trial number), *F* (3.276, 160.510) = 1.848, *p* = .135, η^2^ = 0.036. The percentage of valid data for unthresholded trials was approximately 88% valid data throughout the first four trials, then decreased across the remaining two trials to a minimum of 82.84% of valid data in Trial 6. In comparison, after applying a data quality threshold, 32 participants had all 6 trials meeting or surpassing the 70% threshold. Comparing the percentage of valid data across the 6 trials of video stimuli for these 32 participants, there was no significant loss of data across time, *F* (3.629, 112.493) = 2.034, *p* = .101, η^2^ = 0.062. The percentage of mean valid data in a trial ranged from a maximum of 95.89% valid data in Trial 1 to a minimum of 91.84% valid data in Trial 6.

### Fixation metrics

**Fig 11:**
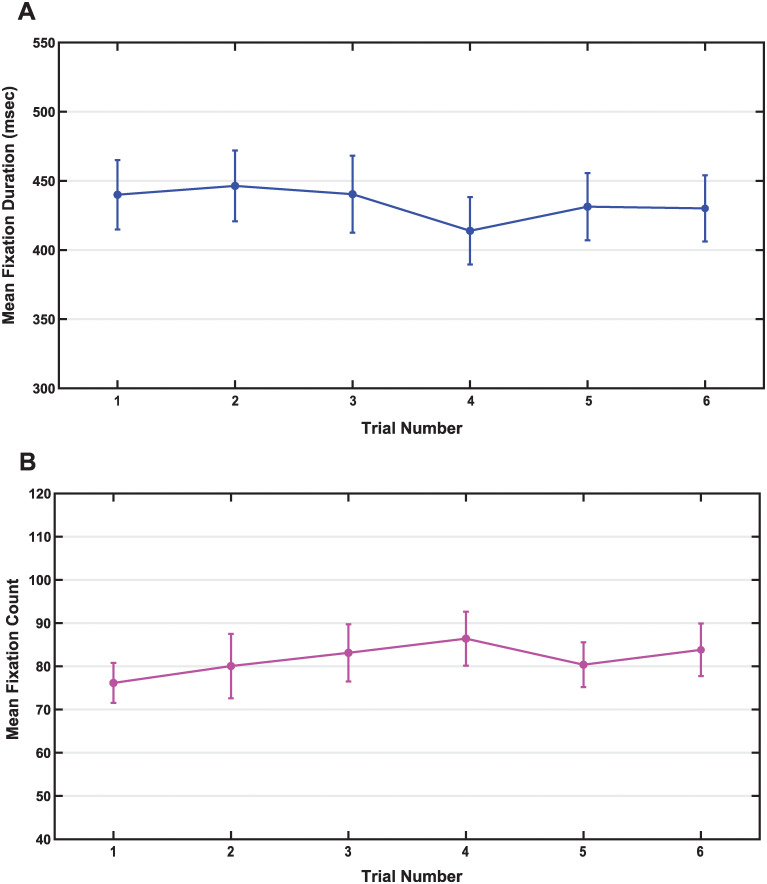
Fixation metrics across time for thresholded data. There were no significant differences in fixation duration (A) or count (B) across the 6 trials of data collected. Data presented as mean +/- 1 SEM.

There were no significant differences in average fixation duration or average number of fixations across time. No differences were found for the average fixation duration across trial number (Fig 11A), *F* (5, 155) = .571, *p* = .722, η^2^ = 0.018. Additionally, mean fixation duration remained stable between the first and last trials (Trial 1: *M* = 439.99 ms, *SD* = 141.97; Trial 6: *M* = 430.11 ms, *SD* = 135.36). Average fixation duration across all trials was 433.68 milliseconds (*SD* = 119.41). Similarly, the average number of fixations made in each trial did not change across trial number (Fig 11B), *F* (3.147, 97.543) = .721, *p* = .548, η^2^ = 0.023. Mean number of fixations remained stable between the first and last trials (Trial 1: *M* = 76.16, *SD* = 26.22; Trial 6: *M* = 83.81, *SD* = 34.47). The average number of fixations across all trials was 81.66 fixations per 50 seconds of trial data (*SD* = 26.87).

### Saccade metrics

**Fig 12:**
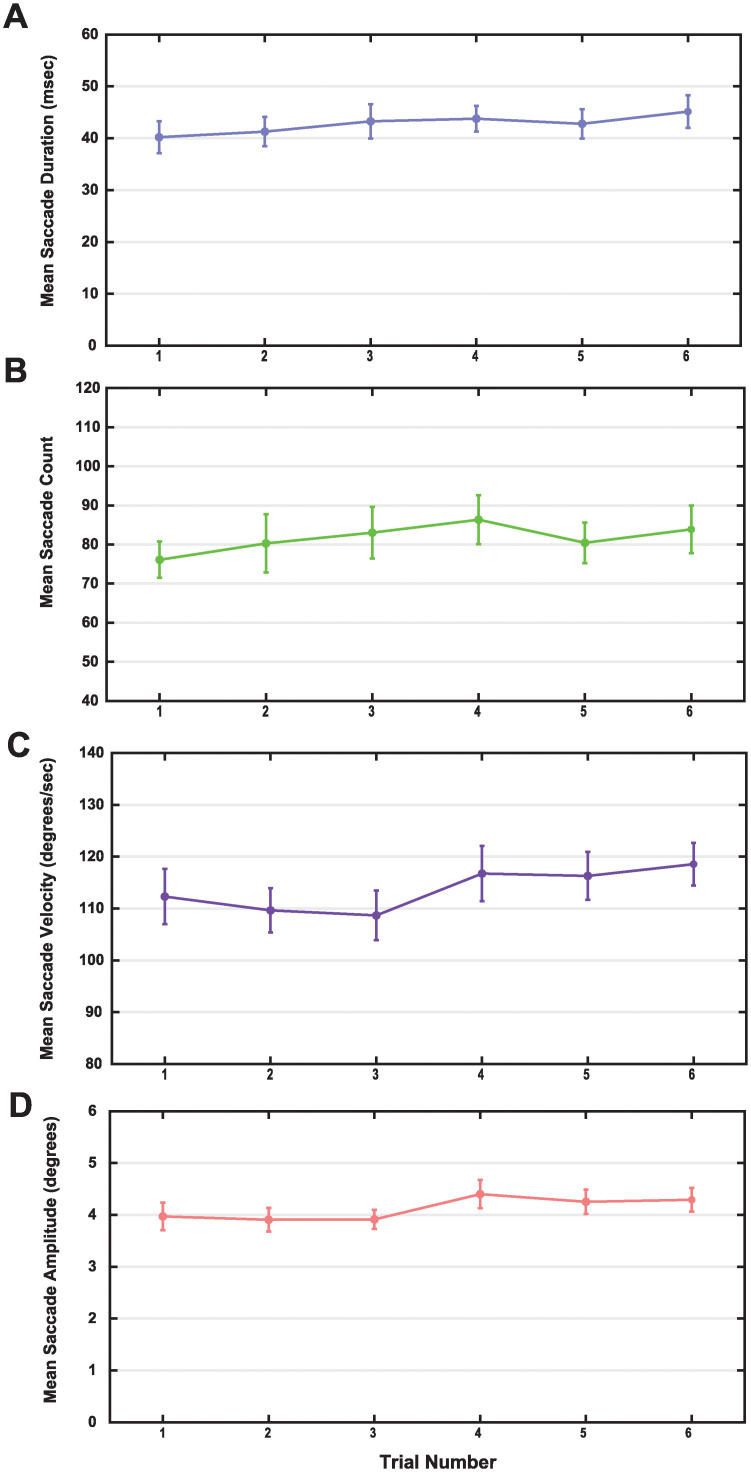
Saccade metrics across time for thresholded data. Saccades did not significantly change in duration (A), count (B), mean velocity (C), or amplitude (D) across time. Data presented as mean +/- 1 SEM.

No significant differences were found for any saccade metrics across time. Saccade duration did not significantly vary across the 6 trials (Fig 12A), *F* (5, 155) = 1.228, *p* = .298, η^2^ = 0.038, (Trial 1: *M* = 40.20 ms, *SD* = 17.51; Trial 6: *M* = 45.13 ms, *SD* = 17.91). The mean across all trials was 42.73 milliseconds per saccade, (*SD* = 14.57). Saccade count did not vary significantly across time (Fig 12B), *F* (3.154, 97.774) = .715, *p* = .552, η^2^ = 0.023, (Trial 1: *M* = 76.10, *SD* = 26.21; Trial 6: *M* = 83.88, *SD* = 34.35). Mean saccade count across all trials was 81.68 saccades per 50 seconds of trial data (*SD* = 26.89). Mean saccade velocity did not vary over time (Fig 12C), *F* (5, 145) = 1.997, *p* = .082, η^2^ = 0.064, (Trial 1: *M* = 112.30 degrees per second, *SD* = 24.58; Trial 6: *M* = 118.55 degrees per second, *SD* = 22.95). Mean velocity across all trials was 113.69 degrees per second, (*SD* = 19.65). Lastly, saccade amplitude remained steady over time, showing no significant change over the 6 trials (Fig 12D), *F* (5, 145) = 2.240, *p* = .053, η^2^ = 0.072, (Trial 1: *M* = 3.97 degrees, *SD* = 1.10; Trial 6: *M* = 4.29 degrees, *SD* = 1.16). Mean amplitude across all trials was 4.12 degrees, (*SD* = 0.78).

## Discussion

This study provides a new strategy for remote eye-tracking collection that utilizes a task that is feasible for infants younger than 3 months of age. Additionally, it is the first to detail oculomotor metrics in infants as young as 2.5 months. Specific adaptations to more common eye-tracking techniques were made to enable reliable and robust data collection in this population of infants, who have minimal neck stability and control. These modifications included 1) application of a simple set up strategy that incorporated the infant being held in a carrier and 2) the use of an audible lost signal track. The carrier and infant neck pillow minimized the loss of corneal reflection that can occur due to positioning challenges such as neck instability and lower lid protrusions. The auditory lost tracker signal allowed the researcher to intervene and remedy a lost signal in real time by changing the positioning of the infant, thereby increasing the probability of collecting usable data across the 6-minute session. Use of this cue is not typically necessary with older participants, who can control their own neck muscles, and whose inattention can generally be observed and redirected if the head turns away from the monitor. As neck control is not fully developed in 2-month old infants and small movements in any direction can impair the ability of the camera to track the eyes even when attention is engaged, the audible signal was critical in notifying the researchers of lost tracking due to participant inattention or positioning and allowed for robust data capture.

An additional modification to the setup was use of a longer-wavelength 940nm illuminator (SR Research), rather than the commonly used 890 nm infrared illuminator. This illuminator was recommended for young infants as the 940nm wavelength light provides higher image contrast for pupil detection of infants’ eyes. The higher wavelength light allows for the camera to better identify the pupil prior to the stabilization/permanence of the infants’ eye pigmentation by 9–12 months of age as blue-grey pigmentation may result in lower accuracy tracking and produce noisier data [19, 48–50]. We also made several simple, logistical modifications to allow for successful data collection. One example was a brief, but very important period of time that was incorporated to allow for the researcher to establish rapport with the participating parent to alleviate concern handling their new baby. This reduced uncertainty and anxiety in the parent and promoted uninterrupted and successful data collection. In addition, flexibility in the timing of implementing the task was essential, to address issues such as breaks for infant sleep or feeding.

In this study, we demonstrate that a 6-minute eye-tracking task is feasible in infants as young as 2.5 months of age. Valid data collection across the video task was consistently very high, ranging from 93% to 96%. Furthermore, we have demonstrated for the first time that infants at this age attend to a variety of stimuli in a video format, which, in comparison to static stimuli, approximates a more realistic viewing setting in which there are competing dynamic stimuli. Furthermore, we demonstrate that 2.5-month old infants maintain consistent fixation and saccade behavior across a 6-minute window of data collection. This allowed us to collect and report precise characterization of oculomotor dynamics for the first time in infants at this age. Visual inspection of the data indicated that to avoid confounding effects from fatigue or inattention of infants, an eye-tracking task should likely not exceed 6 minutes in this early infant age range. The analyses indicate that this time period is more than sufficient to obtain high quality eye movement measures.

The experimental strategy used in this study produced successful data collection. Of the 54 infants enrolled, 50 were awake and compliant and completed the 6-minute task. Of those 50 who completed the task, 32 met or surpassed 70% data quality thresholding. Interestingly, those participants who did not meet or surpass data quality thresholding at 70% were statistically younger than those that did (Table 1). Future exploration of the relationship between age and data quality thresholding will help determine whether different quality thresholding or additional modifications would allow for inclusion of data from infants younger than 2.5 months of age. Prior eye-tracking studies have described how data quality can affect measuring of key output variables such as fixation duration and saccade count [46]. Our data also support these findings: as the percentage of valid data decreases, fixation duration decreases and the number of saccades increase (Fig 8). Data loss can be caused by a variety of factors, most commonly hardware or software malfunction, or changes in head positioning of the infant participant [46, 51]. It is important to note that there is no universally-agreed upon data quality threshold above which eye-tracking data is deemed usable at any age. Data thresholding reported for infant studies has ranged as low as 40%–50% valid data [52] to 80% [53, 54] and in some studies thresholds are not reported at all [55, 56]. Thus, the selection of an appropriate threshold value is still largely subjective and depends on the research questions of the study.

Eye-tracking metrics can assist in characterization of problems in attentional development that influence a multitude of associated domains [57], including executive functioning [58], response inhibition [59], and social and emotional development including self-regulation [25, 27]. Deficits in attention in young children are often difficult to detect prior to formal school settings. Early detection of disruptions in the structure and function of oculomotor movements which feed into these attention systems can identify children at greatest risk and allow for opportunities to provide needed interventions and therapies at an early age. Indeed, such approaches using oculomotor behavior to understand typical and atypical development are already applied to older children [59, 60].

Because gaze behavior patterns can be used to discern diagnostic groups, methodology that is sensitive to differences and can be used in early infancy to identify those at risk for neurodevelopmental disorders over time is needed. Though our sample size is small for reporting normative data, and therefore a limitation of the current study, the methodology established here will allow for more in-depth analysis of atypical neural development that can be detected using oculomotor dynamics within infant populations. With a capacity to perform measures in early infancy, one could determine how aberrations in fixation and saccade data collected in a free-viewing task can be used to detect and characterize the precursors of attentional and developmental disorders such as ASD, ADHD, FASD, and others within infant populations [10–14, 61]. Our methodology will provide opportunities to determine whether foundational oculomotor dynamics captured in a free-viewing naturalistic setting will similarly allow for early detection of a variety of important abnormalities within infant populations, to better identify those at-risk at a very early age, possibly before diagnosis.

The findings here suggest that specific methodological adaptations allowed for the first report of successful collection of eye-tracking data and establishing reproducible oculomotor metrics for 2–3-month-old infants. Furthermore, with continued development of advanced optics in hand-held devices, the strategies used here are simple enough to be used in future projects seeking to expand data collection in scalable ways for pediatric practices. Finally, the flexibility of the eye-tracking system, and straightforward adaptations that can be used by any health or research professional, will allow for similar data collection across sites, across infant development, and in longitudinal studies.

## Supporting information

S1 Dataset Minimal dataset. (XLSX)
